# Biotinidase Deficiency in Newborns as Respiratory Distress and Tachypnea: A Case Report

**Published:** 2015

**Authors:** Shahin KOOHMANAEE, Marjaneh ZARKESH, Manijeh TABRIZI, Afagh HASSANZADEH RAD, Siamak DIVSHALI, Setila DALILI

**Affiliations:** 1 Pediatric Growth Disorders research center, School of Medicine, Guilan University of Medical Sciences, Rasht, Iran; 2 Department of Pediatric, Endocrinology and Metabolism, Guilan University of Medical Sciences, Rasht, Iran; 3 Department of Neonatology, School of Medicine, Rasht University of Medical Sciences, Rasht, Iran

**Keywords:** Biotinidase Deficiency, Biotin, Newborn, Children

## Abstract

**Objective:**

Biotin is a coenzyme composed of four carboxylases. It presents in amino acid catabolism, fatty acid synthesis, and gluconeogenesis. Biotinidase recycles the vitamin biotin. A biotinidase deficiency is a neurocutaneous disorder with autosomal recessive inheritance. The symptoms can be successfully treated or prevented by administering pharmacological doses of biotin. Although, according to neonatal prenatal medicine (2011), a biotinidase deficiency does not manifest during the neonatal period. In this study, we report on a case of biotinidase deficiency in the first week of birth.

**Case Report:**

A 3100 g term boy was born via cesarean section. After 3 days, he was referred to the 17th Shahrivar Hospital with the chief complaint of tachypnea and grunting. Laboratory results revealed that liver and renal function tests, serum electrolytes, and blood indexes except ammonia were all normal. Within few days after the administration of oral biotin, the patient showed dramatic improvement and was discharged. However, within 4 months he was admitted two other times with the complaints of diarrhea and pneumonia. Unfortunately, he expired after 4 months.

According to our results, it seems that clinicians should accurately assess suspicious patients and even assess infants for biotinidase deficiency.

## Introduction

Biotin is a coenzyme composed of four carboxylases. It is commonly present in amino acid catabolism, fatty acid synthesis, and gluconeogenesis (1, 2). Biotinidase deficiency (BD) is an autosomal recessive disorder that originates by a deficiency of the biotinidase enzyme. The incidence of combined (partial and profound) and profound BD were reported as 1 per 60,089 and 1 per 112,271 of live births, respectively (3). Biotinidase recycles the vitamin biotin. Although, BD has a fundamental influence on neurocutaneous organs and as a cause of neurological disorders, it is, however, indicated by symptoms that can be effectively treated or prevented with therapeutic doses of biotin. In addition, untreated BD can be mentioned as an ideal example of an inherited metabolic disorder that can induce major disabilities (3). Although, most BD patients demonstrate metabolic ketolactic acidosis, organic aciduria, and mild hyperammonemia, it is asymptomatic in children without the presence of organic aciduria or metabolic ketoacidosis and the diagnosis of BD should be performed (4-6). Recently, BD is indicated as a major criterion for newborn screening and is universally approved in the United States as well as other countries (3). BD can be detected by newborn screening or prenatal molecular analysis for mutations. Clinicians usually recommend prenatal molecular analysis in patients with previous familial affected child because of the discovery of carrier states (6) . According to neonatal prenatal medicine, BD does not present in the neonatal period (2, 7). However, in this study, we report a case of BD in the first week after birth.

**Fig 1:**
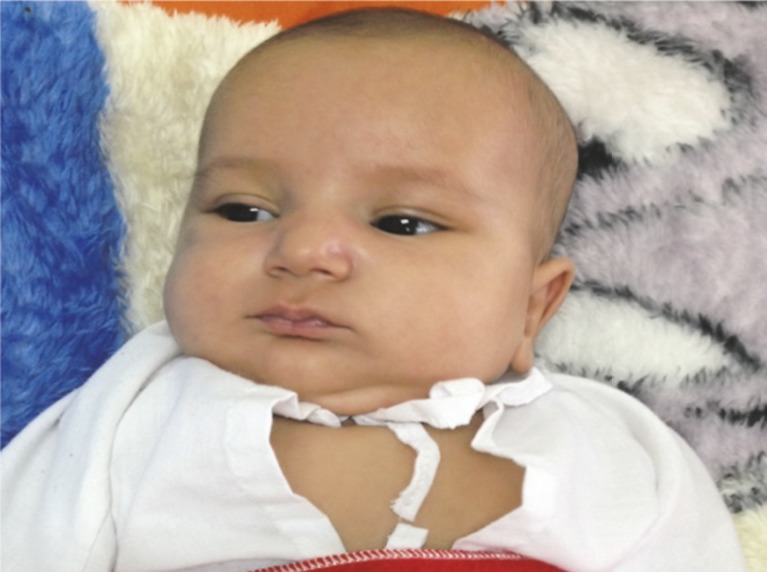
A 3 days old boy who referred to hospital with the chief complaint of tachypnea and grunting

## Case Report

A 3100 gram term boy was born through cesarean section. He was the second born child of consanguineous marriage and did not receive vaccinations at birth. Three days after birth, he was referred to 17th Shahrivar Hospital with a chief complaint of tachypnea and grunting. The anterior fontanel was not bulged and he was conscious but ill during the physical examination. He had grunting and intercostal retraction. A 2/6 systolic murmur at LSB was heard and based on the pediatric cardiologist examination; a heart abnormality was not mentioned. O2 sat = 99%, RR = 58, PR = 156 Laboratory studies revealed that liver and renal function tests, serum electrolytes, and blood indexes except ammonia were all normal (Table 1). In addition, a sepsis workup including blood, urine, and CSF culture had been done and results indicated negative growth. Further, results indicated persistent severe metabolic acidosis based on ABG (pH: 7.01, Po2: 169.4, Pco2: 20.4, Hco 3: 7, and O 2 sat 99 %). In addition, CL and Na had been assessed and a high anion gap metabolic acidosis had been noted and was treated by 40 meq Hco3 (7.5%) or 13 meq/kg/day. According to parental interfamilial marriage and high anion gap metabolic acidosis, the clinicians decided to detect for metabolic disorders such as ammonia, lactate, tendon mass spectrum, and urine organic acid. Further, increased lactate and ammonia was observed. Our results showed abnormalities in the blood and urine. The presence of 3-hydroxyisovalerylcarnitine (C5-OH) and decreased biotinidase enzyme indicated BD. Within few days after administration of oral biotin (10 mg / day), the patient showed dramatic improvement by normalization of blood gas analysis. Therefore, clinicians recommended discharge after 20 days. However, within 4 months, he was admitted two other times with the complaints of diarrhea and pneumonia. Unfortunately, he expired after 4 months.

## Discussion

An infant with early presentation of BD was described. On admission, he had severe neurologic symptoms and by administering continuous biotin showed dramatic improvement. In addition, previous investigations recommended continued biotin therapy (8, 9). Unfortunately, on time and accurate diagnosis and treatments could not prevent further complications and the child expired after 4 months. It seems that it happened because of immunodeficiency combined with BD led to his expiration (10). In this study, we reported BD in the neonatal period. However, Zinn et al mentioned that BD does not manifest in the neonatal period (2), which is inconsistent with our results. Also, Andersen et al noted 2- and 3-weeks as the earliest reported age for BD (7). No complaint of seizure was mentioned in the current study. However, Venkataraman et al mentioned seizure as the presenting complaint in all patients and clonic seizure was the predominant seizure type (11). Moreover, Bunch et al mentioned that seizure was a common feature (55 % in Salbert series) and usually an initial symptom (38 %) in profound BD. They also noted generalized tonic–clonic (56 %) seizures (12). Moreover, in this study the patient had persistent severe metabolic acidosis, which was inconsistent with Venkataraman et al. In addition, Venkataraman et al noted acidosis in no patients (11). Furthermore, in this study, results showed abnormality in both blood and urine 3-hydroxyisovalerylcarnitine (C5- OH) and indicated BD. However, a previous investigation indicated increased blood 3-hydroxyisovalerylcarnitine (C5-OH) in beta-ketothiolase deficiency, 3methylcrotonyl-glycinuria, biotinidase deficiency, 3-hydroxyisobutyric aciduria (13) As decreased levels of biotinidase enzyme by ELIZA in filter paper had been reported and may indicate a BD diagnosis, which should be confirmed by genetic methods.

**In conclusion**, according to our results, it seems that clinicians should accurately assess suspicious patients and even assess infants for BD.

**Table 1:** Results of the laboratory tests

**Laboratory tests**	**result**	**Normal range**
K	5.2	3.4–5.3 mmol /L
Na	137	137–147 mmol /L
Cl	102	99–108 mmol /L
Ca	9.5	8.7–10.7 mg /dL
Lactate	4	0.9–1.8 mmol /L
Ammonia	150	25-80 mmol /L
FBS	85	60–99 mg /dL
BUN	7	5–18 mg /dL
Cr	0.7	0.75–1.20 mg /dL
